# Progress of Diseases of the Throat and Nose

**Published:** 1902-01-25

**Authors:** 


### Progress of Diseases of the Throat and Nose.

(*Continued from page* 273.)

**Malignant Neoplasms.**—Sir Felix Semon[19] recently showed a patient at the Laryngological Society, London, from whom he had removed an extensive malignant growth which had occupied the whole of the right half of the larynx, completely destroying the right vocal cord, and also extending below the anterior commissure to the front part of the subglottic cavity, and attacking the front part of the lower surface of the left vocal cord. Shattock reported that the sections of his removed fragments showed it to be a typical squamous-celled carcinoma. The patient was a male age 47, who had been suffering for several months from hoarseness, the only symptom. At the time of the demonstration, nine months after operation, the patient had a wonderfully good voice, and there was no indication whatever of any recurrence.

Six cases of excision of the larynx are recorded by Harvey,[20] and considering how rarely the complete removal of the larynx is performed, are worthy of careful note. He follows Rölter's description of the operative technique for three of his cases. Three patients made a good recovery, one of whom died after twelve months of acute pneumonia. The other two, by means of an artificial larynx, had useful voices. Two patients died without recovering from operation, and one lived over twelve months. All the cases were operated on for extensive malignant disease of the larynx.

**Collapse of the Alæ Nasi.**—One of the most troublesome complaints to deal with effectually, and one failure to correct which often robs operative treatment of post nasal growths of much of its value, is collapse of the alæ nasi. Walsham[21] has tried various rings, semi-circles, celluloid expanders without avail, though massage has not been without some good result in some of his cases. A new method was suggested to him by a plan spontaneously adopted by one of his patients, viz., rolling up a piece of moist cotton wool into a ball of the size of a small pea, which he poked into his vestibule into the little pit just within the lumen at the angle of bending of the lower lateral cartilage. He thought that he might in place of this cotton wool ball transplant there a ball of the patients' own tissue. A strip of mucous membrane as thick as possible, and about $\frac{3}{16}$ths of an inch in width, was dissected up from the inner wall of the vestibule, leaving the base attached above. The surface of the little pit at the angle of bending of the lower lateral cartilage was next made raw by removing the epithelial layer. The epithelial lining was also removed from the little strip of tissue; the tissue itself was rolled up bandage-wise, and then secured to the rawed surface of the pit by a stitch of the finest fishing-gut passed by a needle through the septum into the opposite nostril and back again. When thus fixed the little roll of tissue pressed out the external portion of the lateral cartilage just enough to prevent the ala during inspiration from falling into contact with the septum. It seemed in this case to have produced a complete cure.

**Epistaxis.**—The causation and treatment of profuse epistaxis in people beyond middle life is discussed by Coates.[22] He records five cases, and shows that in all the sequence of events leading up to the epistaxis was as follows: (1) Long-continued high arterial pressure; (2) some sudden cardiac failure; (3) overfilling of the whole venous system; (4) leakage from an overfilled vein. The indication is to empty the overfilled veins. This cannot be done at once by increasing the heart's action, because its weakness is the essential cause of the trouble, he therefore advises the exhibition of such drugs as nitro-glycerine, erythrol, tetranitrate, or possibly, thyroid tablets.

A peculiar case of very persistent and repeated epistaxis is recorded by Compaired.[23] A man, aged 25, had been subject to severe hæmorrhage from the left nasal passage for five years, and these were becoming more and more profuse. He had been treated for anæmia, hœmophilia, and tuberculosis, and lastly, a malignant growth of the nasal fossæ had been diagnosed. The patient had been bleeding for seven days when Compaired saw him. After it had been temporarily controlled rhinoscopic examination revealed a varicose condition of the internal branch of the spheno-palatine artery on the septum; it was thoroughly cauterised, and the patient cured.

[19] Trans. of London Laryng. Soc., Mar., 1901. [20] Lancet, Sept. 21, 1901. [21] Lancet, Mar. 30, 1901. [22] Ibid., April 20, 1901. [23] Jour. of Laryng., June, 1901; Rev. Hebd. de Lar. d'Ot. et de Rhin., Dec. 8, 1900.

